# Personality Disorders among Patients with Mental and Behavioral Disorders due to Psychoactive Substance Use in a Tertiary Care Center of Nepal: A Descriptive Cross-sectional Study

**DOI:** 10.31729/jnma.6176

**Published:** 2021-02-28

**Authors:** Namrata Mahara Rawal, Monalisha Pradhan, Suman Prasad Adhikari, Pradeep Manandhar

**Affiliations:** 1 Department of Psychiatry, Shree Birendra Hospital, Chhauni, Kathmandu, Nepal

**Keywords:** *personality disorder*, *psychoactive agents*, *substance abuse*

## Abstract

**Introduction:**

Personality disorders is comprised of deeply ingrained and enduring behavioral patterns, manifesting themselves as inflexible responses to a broad range of personal and social situations.” Personality Disorders are frequently occurring among patients with substance use disorders. Co-occurrence of substance use disorders and personality disorder is associated with a greater functional impairment affecting treatment adherence. This study's objective was to find out the prevalence of personality disorders among substance use disorders from the Department of Psychiatry and Mental Health, of a tertiary care center of Nepal.

**Methods:**

A descriptive cross-sectional study was done at the Department of Psychiatry and Mental Health of Shree Birendra Hospital, Chhauni, Kathmandu, Nepal. The ethical approval was approved by the Institutional Review Committee prior to the study. The International Personality Disorder Examination ICD-10 module interview schedule was used to determine personality disorders. There were 100 patients, 86 males and 14 females of age ranging between 18-59 years, from different education levels, socioeconomic statuses, and ethnicities.

**Results:**

Of the total 100 patients, 63% of the patients with substance use disorders were found to have either single 24 (24%) or multiple 39 (39%) personality disorders. The most frequently identified disorders were Emotionally Unstable Borderline Type 34 (34%), Anxious Personality Disorder 27 (27%), Emotionally Unstable Impulsive Type 27 (27%).

**Conclusions:**

Present study indicates that personality disorders were highly comorbid with patients of substance use disorders with either single or multiple personality disorders. Personality disorders mostly found in substance use disorders are Emotional Unstable Borderline Type, Anxious Personality Disorder, and Emotional Unstable impulsive Type.

## INTRODUCTION

Personality disorders is comprised of deeply ingrained and enduring behavioral patterns, which are inflexible to a broad range of personal and social situations.” It is a way of thinking, feeling, and behaving that deviates from the expectations of the culture, causes distress or problems functioning, and lasts over time.^1^ The disordered personality traits are associated with high rates of separation, divorce, and unemployment.

A personality disorder is frequently comorbid with substance use disorders. Co-occurrence of substance use disorders and personality disorder is associated with greater functional impairment, indicating a need for increased focus on creating effective treatments to meet the needs of these patients.^2,3^

Personality disorders have been identified as one of the most important predictors of treatment outcome in psychoactive substance users. Therefore, this research was conducted to explore personality disorder types among psychoactive substance users coming to the Department of Psychiatry and Mental Health, Shree Birendra Hospital, Chhauni, Kathmandu.

## METHODS

A descriptive cross-sectional study was carried out among substance use disorders from the Department of Psychiatry at Shree Birendra Hospital. The total period taken for the present study was of one year, from October 2018 to October 2019. The Institutional Review Committee, Nepalese Army Institute of Health Sciences, approved the research protocol prior to the data collection. Participants who came to inpatient and outpatient services in the Department of Psychiatry and Mental Health, Shree Birendra Hospital, were selected after the detoxification. The usual length of treatment at the detoxification is approximately 10-15 days. Many detoxification patients may struggle with abstinence pains, withdrawal symptoms, and other physical problems due to recent substance use, impacting their functioning. Therefore, those stable and completely aware patients after the detoxification period are only allowed to participate in research. They were then informed about the study, and consent was taken.

Sample size was calculated using the formula:


$$\begin{array}{ll} \text{n} & ={\text{Z}}^{\text{2}}\times \text{p}\times \text{(1}-\text{p)}/{\text{e}}^{\text{2}}\\ & =1.96^{2}\times (0.5)\times (1-0.5)/{\left(0.07\right)}^{\text{2}}\\ & =100 \end{array}$$

Where,

n = required sample size
z = 1.96 at 95% Confidence Interval
p = prevalence of personality disorder among psyhchoactive substance users i.e. 15%
e = margin of error, 7%

Convenient sampling was used, consisting of 100 psychoactive substance users who come for outdoor and inpatient service at Shree Birendra Hospital irrespective of age, sex, and psychoactive substance use.

Semi-Structured Interview Schedule (SSIS) and International Personality Disorder Examination (IPDE) were used for data collection. IPDE is a self-ICD-10 item written at a 9-10 year-old reading level. The patient responds either True or False to each item and can complete the questionnaire in 15 minutes or less. The clinician can quickly score the questionnaire and identify those patients whose scores suggest the presence of a personality disorder; therefore, complete IPDE is assessed.

Statistical Package for Social Sciences version 20 was used to input the collected data. Descriptive analysis was done.

## RESULTS

Among 100 patients, 86 (86%) were male, and only 14 (14%) were female. 31 (31%) patients are of age between 18 to 20 years, 35 (35%) are of age between 30 to 39, 14 (14%) are of age between 40 to 49, and 20 (20%) of patients are 50 or above. Further division of age and sex of patients are along with the other demographic characteristics of participants like education, religion, ethnicity, marital status and types of family, are shown below (Table 1).

**Table 1:** Demographic characteristics of participants.

Characteristics	Frequency	Male	Female
Age group	n (%)	n (%)	n (%)
18 to 29 years	31 (31)	25 (25)	6 (6)
30 to 39 years	35 (35)	31 (31)	4 (4)
40 to 49 years	14 (14)	11 (11)	3 (3)
50 and above	20 (20)	19 (19)	1 (1)
years			
Education			
Primary	32 (32)	25 (25)	7 (7)
Secondary	39 (39)	37 (37)	2 (2)
Higher Secondary	24 (24)	20 (20)	4 (4)
Bachelor	5 (5)	4 (4)	1 (1)
Religion			
Hindhu	95 (95)	85 (85)	10 (10)
Buddhist	5 (5)	4 (4)	1 (1)
Muslim			
Ethnicity			
Brahmin	10 (10)	10 (10)	
Chhetri	55 (55)	48 (48)	7 (7)
Newar	4 (4)	4 (4)	
Other	31 (31)	24 (24)	7 (7)
Marital Status			
Married	92 (92)	81 (81)	12 (12)
Single	7 (7)	4 (4)	3 (3)
Types of Family			
Joint	74 (74)	65 (65)	9 (9)
Nuclear	26 (26)	21 (21)	5 (5)

The demographic characteristics of the sample of the study consisted of education, religion, ethnicity, marital status, and family types among patients with a substance disorder. The age of the subjects in each of the two groups, male and female, ranged between 18-59 years of age. Regarding the educational level of the subjects, 39 (39%) of them were in the secondary level, 32 (32%) were in primary level and 24 (24%) of them had completed their higher secondary level education and 5 (5%) completed bachelor level. Among religions, 95 (95%) follow Hindu and 5 (5%) follow Buddhist. Among ethnicity, 55 (55%) of participants are Chhetri, 10 (10%) brahmin and 4 (4%) are Newar. Among the participant, 92 (92%) are married and 7 (7%) are single. Similarly, 74 (74%) live in a joint family and 26 (26%) live in a nuclear family.

It was found that 34 (34%) have EUBT personality disorder followed by anxious 27 (27%), EUIT 27 (27%), dissocial 12 (12%), Anankastic 11 (11%), paranoid B (8%) and schizoid 7 (7%). These are the main personality disorders. The detail of different personality disorders according to the sex of patients and mean age are shown below (Table 2).

**Table 2:** Frequency and profile of personality disorders about gender, age and types of substance use.

Personality Disorder	Male n (%)	Female n (%)	Age (Mean±SD)
Paranoid	6 (6.97%)	2 (28.57%)	50±10.61
Schizoid	7 (8.14%)	-	42.5±14.61
Dissocial	11 (12.79%)	1 (14.28%)	40.53±15.19
EUBT	26 (30.23%)	8 (57.14%)	41.24±14.26
Histrionic	2 (2.32%)	-	34±22.63
Anankastic	10 (11.63%)	1 (14.28)	39.91±10.01
Anxious	23 (26.74%)	4 (28.57%)	36.37±10.28
Dependent	1 (1.16%)	-	50±0
EUIT	20 (23.26%)	7 (50%)	40.93±15.09

Source: Field Study, 2018/19

Note: Percentage of total is based on n=100, percentage of male patient is based on m=86, percentage of female patient is based on f=14, percentage of single patient is based on s=7 & percentage of married patient is based on mp=93.

Among the patients, 37 (37%) do not have any personality disorder and the remaining 63 (63%) have at least one type of personality disorder. 24 (24%) patients have a single personality disorder, 23 (23%) patients have two personality disorders, 11 (11%) patients have three personality disorders, 3 (3%) patients have four personality disorders and 1 (1%) each patient has five personality disorders and nine personality disorders. Therefore, 39% of patients have multiple personality disorders. The following table shows the number of personality disorders found in patients according to sex (Table 3).

**Table 3:** Co-occurence of Personality disorders among Substance Use.

Personality Disorder	Total n (%)	Male n (%)	Female n (%)
None	37 (37%)	35 (40.69%)	2 (14.28%)
Single Personality Disorder	24 (24%)	21 (24.42%)	3 (21.43%)
Two Personality Disorder	23 (23%)	16 (18.61%)	7 (50%)
Three Personality Disorder	11 (11%)	9 (10.47%)	2 (14.28%)
Four Personality Disorder	3 (3%)	3 (3.49%)	
Five Personality Disorder	1 (1%)	11 (12.79%)	
Nine personality disorder	1 (1%)	1 (1.16%)	
Total	100 (100%)	86 (100 %)	14 (100%)
Mean	1.29		
Standard Deviation	1.43		

Source: Field Study, 2018/19

Note: Percentage of male patients is based on m=86 and percentage of female patients is based on f=14.

It was found that 61% have an Anxiety personality disorder, followed by 10% psychosis nos (10%), alcohol withdrawal with seizure (9%), psychosis (7%). These are the major diagnosis types found among substance users. Besides that, 5% have Conduct with psychosis and depression/suicide, followed by psychotic disorder (4%) and psychosis with seizure, OCD and Suicidal (3% each), 2% each have suicidal, ATPD, deliberate self-harm, depressive disorder, hypertension, PDD, Suicidal/BPD and HTN, and 1% each have a panic attack and somatoform disorder. It is also found that 17 patients (17% from total patients) who suffer from anxiety disorder have a single type of personality disorder, 10 patients (10% from total patients) who suffer from anxiety disorder have two types of personality disorder and five patients (5% from total patients) who suffer from anxiety disorder have three types of personality disorder.

## DISCUSSION

Participants of the study only represent substance dependence of Shree Birendra Hospital, which is the limitation of the study. Findings may not be generalizable to the entire Nepalese population because the general people do not have access to the hospital for treatment.

## CONCLUSIONS

Our study indicates that personality disorders were highly comorbid with patients of substance use disorders with either single or multiple personality disorders. Personality disorders mostly found in substance use disorders are Emotional Unstable Borderline Type, Anxious Personality Disorder, and Emotional Unstable impulsive Type.
